# Occupational Stress Monitoring Using Biomarkers and Smartwatches: A Systematic Review

**DOI:** 10.3390/s22176633

**Published:** 2022-09-02

**Authors:** Analúcia Morales, Maria Barbosa, Laura Morás, Silvio César Cazella, Lívia F. Sgobbi, Iwens Sene, Gonçalo Marques

**Affiliations:** 1 Graduate Program in Energy and Sustainability, Sciences, Technologies, and Health Education Center, Federal University of Santa Catarina (UFSC), Araranguá 88906-072, Brazil; 2 Research Group on Intelligent Systems Applied to Health, CNPq, Brasilia 70067-900, Brazil; 3 Graduate Program in Information Technologies and Health Management, Department of Exact Sciences and Applied Social, Federal University of Health Sciences of Porto Alegre (UFCSPA), Porto Alegre 90050-170, Brazil; 4 Institute of Chemistry (IQ), Federal University of Goiás (UFG), Goiânia 74690-900, Brazil; 5 Institute of Informatics (INF), Federal University of Goiás (UFG), Goiânia 74690-900, Brazil; 6 Polytechnic of Coimbra, ESTGOH, Rua General Santos Costa, 3400-124 Oliveira do Hospital, Portugal

**Keywords:** pulse wearable, physicochemical parameters, stress measurement, Internet of Things

## Abstract

This article presents a systematic review of the literature concerning scientific publications on wrist wearables that can help to identify stress levels. The study is part of a research project aimed at modeling a stress surveillance system and providing coping recommendations. The investigation followed the Preferred Reporting Items for Systematic Reviews and Meta-Analyses (PRISMA) guidelines. In total, 38 articles were selected for full reading, and 10 articles were selected owing to their alignment with the study proposal. The types of technologies used in the research stand out amongst our main results after analyzing the articles. It is noteworthy that stress assessments are still based on standardized questionnaires, completed by the participants. The main biomarkers collected by the devices used in the selected works included: heart rate variation, cortisol analysis, skin conductance, body temperature, and blood volume at the wrist. This study concludes that developing a wrist wearable for stress identification using physiological and chemical sensors is challenging but possible and applicable.

## 1. Introduction

Every day, health professionals suffer from work-related stress, which can lead to physical, emotional, or mental problems, and consequently have a negative impact on patient care and increase public health expenses. Occupational stress has worsened in recent years given the overload on health systems since the emergence of the COVID-19 pandemic in Wuhan, China. Among the physical symptoms pointed out by professionals in the care of severe acute respiratory syndrome infections (SARS), the following stand out: fever, headaches, myalgias, chills, nausea, vomiting, and diarrhea. In addition, the most frequently reported mental disorders were mapped: psychological distress, anxiety, insomnia, fear, depression, and burnout [1]. The authors of ref. [2], state that to prevent stress from becoming chronic and causing irreversible damage, it is necessary to detect the problem in its early stages, when continuous, automatic, and discrete monitoring must be taken into account, considering the multimodal nature of stress. Owing to subjectivity and variability between individuals, occupational stress involves several variables, together with physical, physiological, emotional, and psychological changes; thus, measuring stress is a challenging task. However, given technological advances and the emergence of sensors capable of capturing physiological states, state-of-the-art smartwatches, in addition to intelligent computing resources, make it possible to monitor various aspects of human routine, including behaviors related to the individual’s specific stress conditions, in a personalized approach [3].

Stress can have physiological, behavioral, or psychological manifestations. One of the physiological responses of the human body is changes in metabolism, manifested through the release of hormones, such as cortisol and adrenaline. The cortisol level cycles throughout the day, starting at high levels and gradually decreasing until its lowest level is reached during the night [3], when the production of melatonin begins. However, individuals with chronic stress maintain high levels of cortisol throughout the day, including at night, which compromises sleep and generates emotional imbalances and mental disorders, such as depression, lack of motivation or purpose, anxiety, fear, and irritability [4]. People with high stress levels tend to suffer from insomnia and may experience concentration issues and mood swings, directly interfering with their professional activity. Furthermore, high cortisol levels may be responsible for gastrointestinal diseases such as gastritis, ulcer, and cancer [5]. However, cortisol, which can be measured by sensors using saliva samples or smartwatches, is one of the biomarkers that has been researched and identified as promising in this new technological context, which involves the Internet of Things (IoT) applied to health [5,6].

Despite the impacts and technological advances, the evaluation and diagnosis process is highly complex, as it involves physical, mental, and emotional health. Overall, stress can still be divided into two types: acute and chronic. Acute stress results from a specific event or situation; it is short-lived and can be accompanied by physical symptoms such as rapid heartbeat, sweating, and headaches, but it can also generate motivation toward dealing with stressors. Acute stress should not be exclusively associated with negative issues, since it can also enable a productive response by the organism, in the face of a specific stimulus, such as a promotion in the work environment or any source of intense excitement and happiness [7]. On the other hand, chronic stress can result in all the diseases mentioned above. The signs can be seen in the form of symptoms such as excessive sweating, chills, body aches, stomach upsets, dry mouth (xerostomia), cold extremities, rapid heartbeat, wheezing, tremors, hypertension, and intestinal disorders. Other consequences can be manifested in the mind through insecurity, insomnia, anguish, hopelessness, fear, and panic, among others. When associated with work stressors, the issues range from loss of productivity and work accidents and will increase health costs and absenteeism from work [8]. The influence of psychosocial factors and work-related organization on health has been the subject of increasing study, and these factors indeed have a significant health impact on the workers, especially on their well-being and quality of life [9]. It is relevant to highlight that work-related stress does not disappear when the worker arrives home after working hours. Health professionals can deal well with adaptations and moments of stress, but tolerating long periods under these conditions without any type of illness (physical or mental) is challenging and can cause consistent health damage, absence from work, and a chronic stress condition. Therefore, a system that monitors affective states can contribute to the early detection and treatment of mental and emotional disorders in healthcare workers, thus preventing them from evolving into a burnout situation [10]. 

Research advances in IoT applied to healthcare (healthcare IoT—HIoT) have contributed to a significantly higher interest in sensor technology in recent years. Specifically, the industry of sensors and flexible materials for healthcare has been extensively explored. The main challenge has been integrating real-time monitoring of physiological parameters through IoT resources in uncontrolled environments. In recent years, several commercial devices in different areas have been developed by pharmaceutical and electronic companies, such as noninvasive digital glucose meters and heart rate monitors or implantable devices able to dosage patients’ medicaments [11]. Wrist wearables (or smartwatches) have gained popularity due to their monitoring capabilities for physical activities, allowing quick access to vital information while employing communication networks through the Internet to transmit and store the information in the cloud and allow the data usage to improve the performance from the users [12]. 

HIoT is a paradigm that presents numerous progress possibilities for this area, interconnecting a series of devices, together with processing and synchronizing data with the responsible professionals, which enables remote monitoring and assistance, in addition to providing its users greater control over their lives and over the treatment itself. In addition, when associated with artificial intelligence techniques, these resources have great potential for the development of systems that can monitor individuals’ physical and emotional states, whether patients or health professionals [13]. Several research studies have reported effective results in the use of machine learning and deep learning techniques associated with the HIoT paradigm, due to their multimodal characteristics and the volume of generated data. Such studies range from applications in highly complex diagnostic processes (different types of cancer and heart diseases), medication management, monitoring patients with chronic diseases and elderly care, patient rehabilitation processes, to different types of medical decision-making [14,15,16].

The identification of stress is based on the application of self-report scale questionnaires. Some samples follow [17,18]:

- DASS-21: A stress assessment instrument composed of 21 questions, which in many cases may be adapted to specific populations. Response scores are classified into five stress levels: normal, mild, moderate, severe, and extremely severe. In addition, this technique provides in the same questionnaire different outcome punctuations for depression and anxiety.
- Perceived Stress Scale (PSS): A classical instrument for stress assessment. The answers present three levels of score stress classification: low stress, moderate stress, and high perceived stress.
- Coping Inventory for Stressful Situations (CISS): A 48-item measure comprising a three-scale task, emotional and avoidance.

The stress may be positive (eustress) or negative (distress). The reaction of the body is the same to both conditions. Physiological reactions have been accompanied by emotional disorders and generate insomnia, lack of concentration, loss of appetite, and other stress conditions related to environment and situations. This work is the first step of a research project that aims to develop an occupational stress surveillance model for health professionals. From the preliminary results presented in this systematic review on wearable wrist devices, it will be possible to understand the use of different technologies, identify the main biomarkers that have been used in related works, characterize the types of stress considered, assess the research-related time, and, consequently, evaluate techniques that can be associated between wearables and other technologies used in occupational stress assessments.

The paper is organized into five sections. The Section 2 presents the method of the systematic review, research gaps, search strategies, and the selection procedures and exclusion criteria used for articles. Section 3 presents a summary of the selected articles, and Section 4 analyzes these results by addressing the points of interest in the related research project. The latter is followed by the final considerations and future work.

## 2. Methods

The review was based on Preferred Reporting Items for Systematic Reviews and Meta-Analyses (PRISMA) guidelines [19]. The objective of the study was to fill a few research gaps regarding the use of smartwatches to assist in the early identification of stress conditions in health professionals, especially those who work on the front lines of COVID-19.

Such gaps were specified through the following research questions:

(RQ1) What methods, techniques, and architectures have been employed in investigations related to the use of smartwatches to monitor stress?
(RQ2) Which stress levels are used in the investigations?
(RQ3) What are the measured data? Are they physiological, mental, or emotional measurements?
(RQ4) What AI techniques are applied to the stress ratings? (Where relevant).

### 2.1. Adopted Criteria and Selection Procedures

The search process was performed using the PubMed Central, VHL (Virtual Health Library), PsycInfo, IEEE Xplore, and Nature electronic databases. Scientific articles were found in the literature in English, Portuguese, and Spanish, observing the use of wearable devices by health professionals. A first evaluation was carried out, based on the titles, abstracts, and keywords of each article. The articles that did not meet the inclusion criteria were withdrawn from the study. When there was uncertainty in the first evaluation, the full text was analyzed in a second evaluation. The criteria applied in the selection of all articles are described in Table 1:

### 2.2. Selection Process

**Figure 1:**
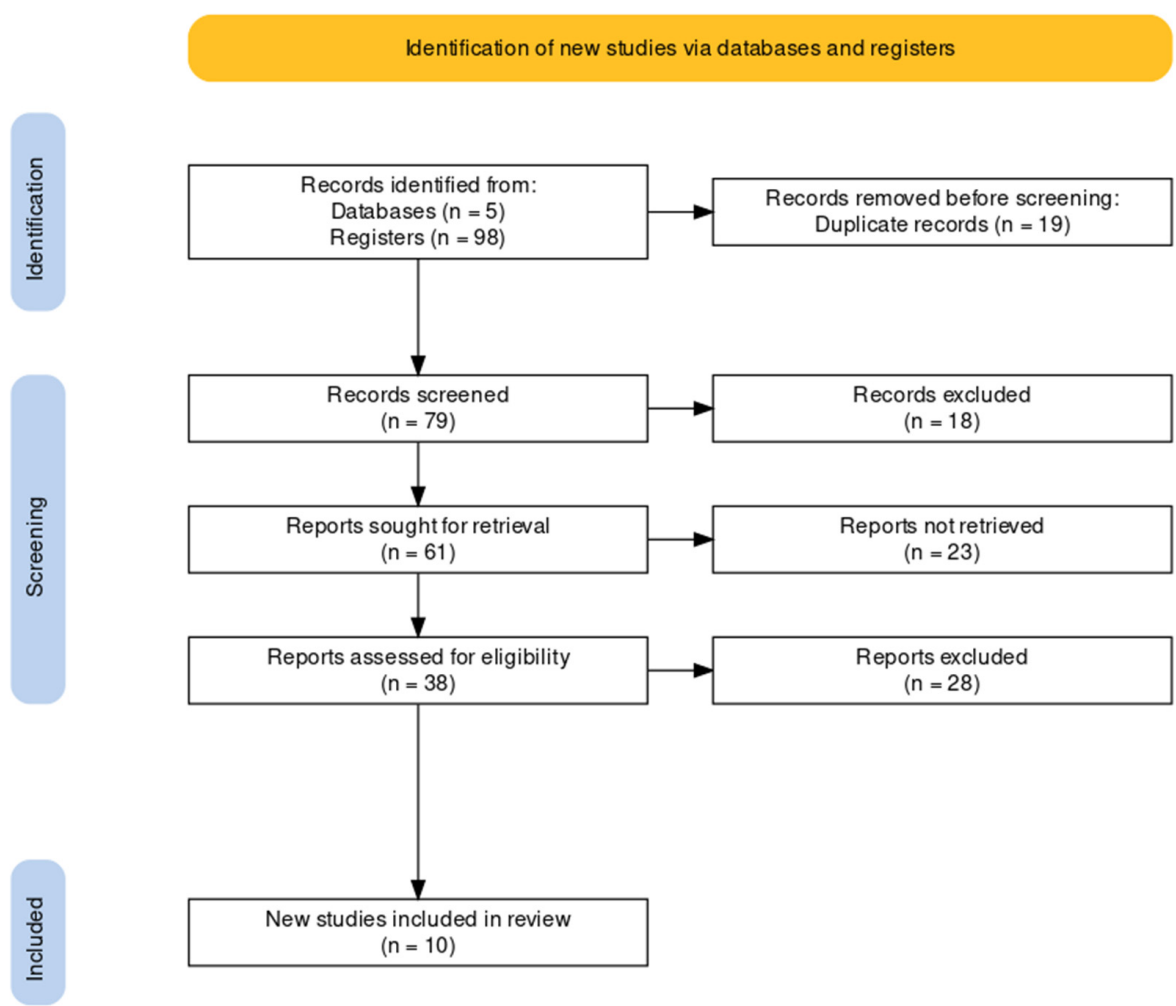
PRISMA flow diagram for systematic review of wrist wearables to identify stress.

The selection started with 98 articles: 29 from PUBMED, 12 from the VHL, 1 from the PsycoInfo database, 1 from the IEEE Xplore, and 28 from Nature, in addition to 13 articles obtained from other bibliographic references of identified articles. From the 98 articles, 19 were excluded due to duplication. Of the remaining 79 articles, 41 were excluded because they did not meet the selection criteria. Thirty-eight articles were selected for full reading, and, in the end, 12 articles remained. Finally, from 12 articles, two were excluded because the focus was on investigating pregnancy stress. Ten articles were selected that were aligned with the research objective. Figure 1 presents the PRISMA diagram of the systematic review, which illustrates the eligible investigations process. For all investigated databases, a two-part search string was used: “smartwatches” AND “stress”. No papers were excluded based on quality assessment; this review aimed to explore the state of knowledge in the field by investigating the monitoring and identification of stress levels in different applications and the technological approaches to solving problems.

### 2.3. Selected Articles

Of the ten selected studies, only one presents the development of a wrist wearable to investigate stress through the detection and analysis of cortisol from small volumes of sweat using a portable platform in the form of a watch [6]. The studies reported in Refs. [10,20,21,22,23,24,25,26,27,28] propose data collection devices combined with machine learning techniques or deep learning. These studies include data collection of physiological parameters for monitoring and identification of stress. The authors of [20] propose an application for occupational stress in health professionals that uses the method of detection of continuous voltage based on smartwatches using individual classifiers and sets of classifiers. The objective is to allow hospital workers the ability to monitor their stress levels. The results of the experiment show that all classifiers worked very well to detect stress with an accuracy of more than 70%.

### 2.4. Distribution of Articles by Publication Date and Location

**Figure 2:**
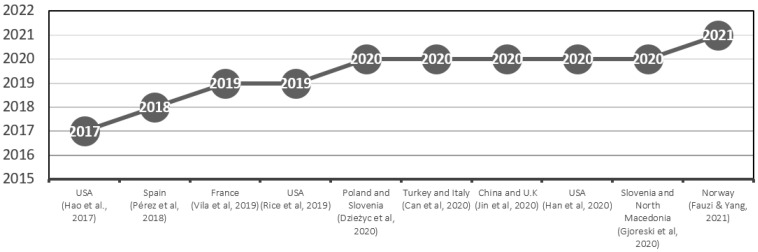
Synthesis of distribution of included papers considering a timeline and their countries. The X-axis represents country and reference, and Y-axis the publication year. The dots indicate the publication year [6,10,20,21,22,23,25,26,27,28].

Included in the review after the application methodology are the reported researchers’ various nationalities: USA [6,23,27], Poland and Slovenia [10], Norway [20], Spain [21], France [22], Turkey and Italy [25], China and UK [26], and Slovenia and North Macedonia [28]. The synthesis of distribution considering the year of publication is plotted in Figure 2.

**Figure 3:**
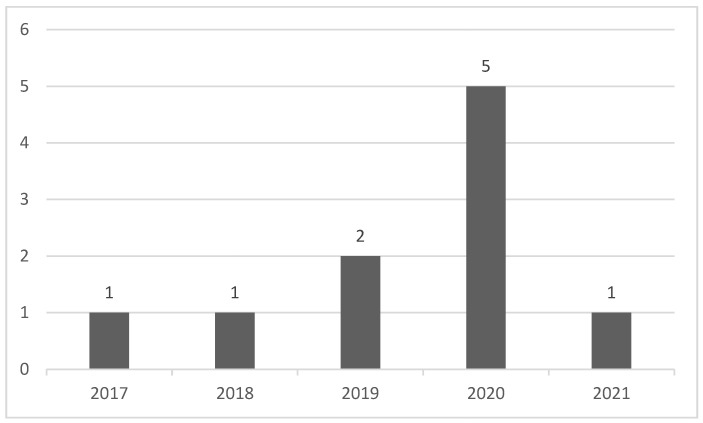
Selected papers per-year distribution.

The selected papers were published from 2017 to 2021; there were five works in 2020, demonstrating a gradual yearly increase concerning this theme to 2020; after this year, curiously, only one work was matched. The distribution per year is illustrated in Figure 3.

Most stress analysis studies apply questionnaires to their participants. However, this is not a viable technique for continuous monitoring involving daily activities, especially in the case of health professionals [20]. There are several standard questionnaires that are filled in by the participants using different scores to identify health workers’ stress after the pandemic as suggested by refs. [17,18].

Regarding the time, the wearable technology was actively used, results varied between 50 and 70 min [21], 1 day [22], 7 days, and 29 days [23]. To consider stress as chronic and harmful to health, causing physical, emotional, and mental disorders, a longer duration of time was considered [24]. Table 2 summarizes the data collection time from the selected works. Within the selected articles, a great potential can be observed in using smartwatches to assist in the real-time identification and control of stress. Specifically, there is an interest in occupational stress focused on health professionals, and only one article was considered in this context [20]. The studies are distributed into occupational stress (20%), stress in academic and cognitive activities (40%), stress in other activities (20%), stress based on the identification of emotions (10%), and stress based on hormone identification (10%). 

One of the main challenges in stress identification is to develop a model that accurately classifies the stress levels using wearable technologies, such as smartwatches. About 90% of the studies present the identification of stress based on physiological signs, as shown in Table 3. Only one study assesses stress through cortisol [6].

## 3. Results

The main analyzed information in the selected articles aims to answer the research questions that guide this systematic review: communication protocols used in the system, type of sensor, biomarkers collected by the sensor, applied machine learning or deep learning techniques, measurements/metrics for the evaluation of results, and standardized stress levels for the analysis of participants. Table 3 summarized the results that help with the answers to the four questions RQ1, RQ2, RQ3, and RQ4, which will be discussed in the following sections.

### 3.1. Answer RQ1

**Figure 4:**
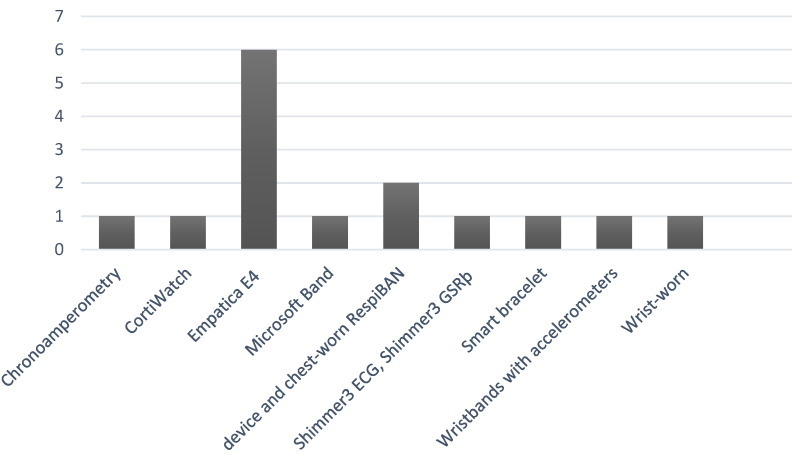
Different sensors were employed in the selected work. The X-axis corresponds to the type of wrist wearable in the works investigated. The Y-axis shows the number of times the device was encountered in the research.

Regarding wearable technologies, the following were used: Microsoft Band (10%); Cortiwatch (10%), which aims to identify stress through the detection and analysis of cortisol from small volumes of sweat on the wrist, in addition to the ARM-Cortex4 smart bracelet, with DSP function; model STM32F405 with 6D sensor system (MPU6050) and (SI7021), which aims to measure activity (movement), skin conductance, accelerometer and body temperature (10%). Half of the studies (50%) used the Empatica E4 device for data collection, which incorporates biomarkers for skin temperature, motion-based activities (accelerometers), electrodermal fluctuating, and blood volume pulse on the wristband. Noting that two articles use the results of a dataset called WESAD that presents results from 15 people who were measured with the mentioned device. In one of the articles, it was not possible to identify the type or brand of device used, only the biomarkers that were analyzed [21]. Figure 4 presents the number of times each wearable technology was found in the analyzed studies.

Considering the devices and the form of data transmission, 70% (considering the two datasets that are based on Empatica E4) use Bluetooth, which implies the need to transmit these data through a smartphone or other device that connects the smartwatch with the data storage cloud (Empatica cloud). 

### 3.2. Answer RQ2

Real-time occupational stress assessments employing biomarkers present many challenges [29,30], especially for health professionals, since there are several stress factors, specifically considering their overload in pandemic times or in other infectious disease outbreaks [1,31]. Amongst the technical issues associated with the complexity of the problem, we can highlight the multimodal nature of the information collected from the sensors (various types of data with different characteristics, for processing and analysis), the volume of data to be analyzed, and the frequency with which these data need to be collected to make any sense in the analysis, transmission delays, power outages or sensor battery drainage, and finally, data security and privacy issues, which cannot be discarded.

**Figure 5:**
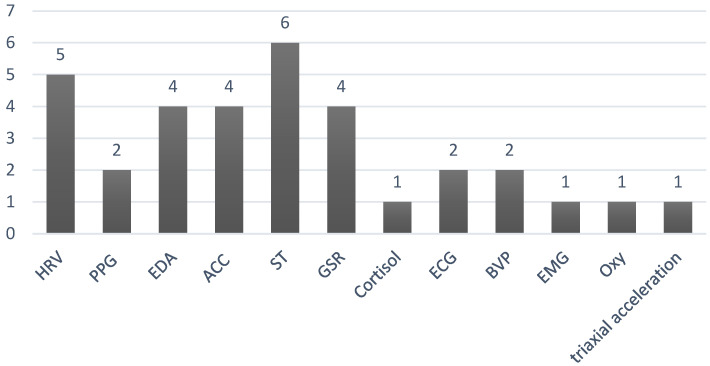
The biomarkers parameters that were mentioned in the selected studies. The X-axis corresponds to the physiological and chemical parameters; the abbreviation list is provided in the appropriate section at the end of this paper. The Y-axis presents the number of times that each parameter appeared in the papers investigated.

The physiological characteristics used in the selected research are similar due to the types of sensors that are currently available on the market. Most articles (90%) use biomarkers that can be collected by smartwatches, and the most frequent are: heart rate variability (HRV, 50%), electrocardiogram (ECG, 20%), skin temperature (ST, 60%), accelerometers (ACC, 40%), electrodermal activity (EDA, 40%), blood volume pulse sensors (BVP, 20%), GSR (galvanic skin rate, 40%), photoplethysmogram (PPG, 20%), Oximeter (Oxy, 10%), and three-axis acceleration (10%). The cortisol chemical parameter was found in only one research work with the identification of two stress-level results. Figure 5 illustrates the frequency of monitored parameters in the selected papers.

**Figure 6:**
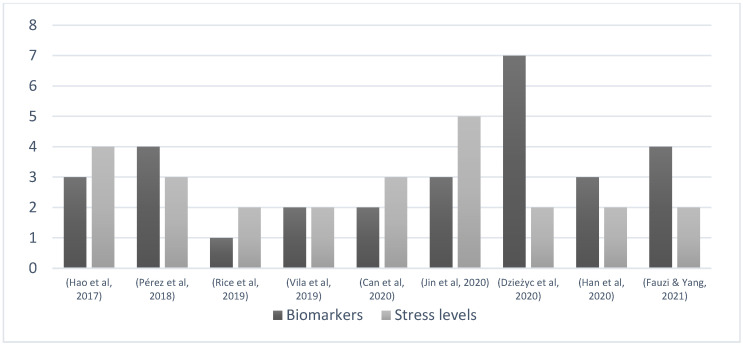
Parameters and stress-level recognition [6,10,20,21,22,23,25,26,27].

The combinations of these parameters are highly relevant for the advancement of stress identification research, as shown by the results of the ten selected studies. The combination of the physiological parameters and emotional investigations is vital to improving the development of wrist wearables for this purpose. However, the complexity of recognizing different categories of stress levels still is a challenge. There is no set of personal physiological parameters reported in the selected papers that can be used to classify the different levels of individual stress situations. To answer RQ2 about the stress levels that were used in the investigations, 50% employed two-level stress in their investigations—presence or absence of stress identification [6,10,20,22,27]. Two present three-level stress identification (20%) [21,25], one work employs four-level stress recognition (10%) [23], and one work presents five-level stress recognition (10%) [26]. The study proposed by [28] does not specify the stress-level considerations in the reported investigation. Figure 6 presents the relationship between biomarkers monitored and the number of stress-level recognition encountered in the selected papers.

### 3.3. Answer RQ3

**Figure 7:**
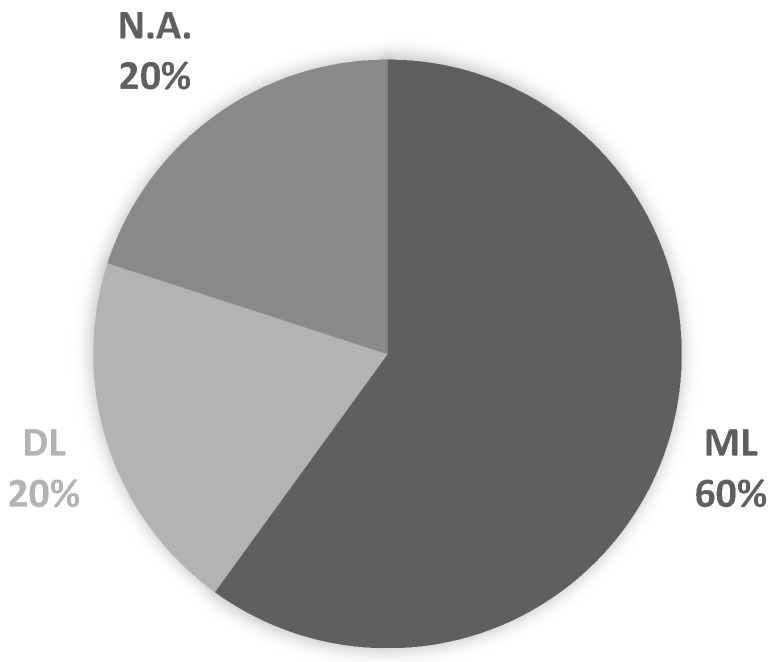
Use of machine learning (ML) and deep learning (DL) techniques in the selected studies. N.A. corresponds to works that did not employ artificial intelligence techniques.

Most of the selected papers employed physiological markers in their investigations (90%), and one used a chemical parameter, cortisol (10%), as shown in Figure 7. The nature of stress and its consideration in the selected investigations in most of the works were based on physiological signs. In two papers, the authors applied emotional questions in their works, [10,26], combining the emotional and physiological parameters in their experiments, both working in multimodal features in their models. None of the articles mentions the assessment of individuals’ mental conditions.

### 3.4. Answer RQ4

In total, 80% of the selected studies that presented results from machine learning or deep learning were based on accuracy metrics for their outputs. The identification of the stress or nonstress situation, which is considered a classification problem in terms of machine learning, was the strategy used by 50% of the studies (two levels). Three levels of stress that varied among low, medium, and high stress were used in 20% of the studies, and one of the studies presented four levels, using less relaxed, relaxed, slightly stressed, and stressed (10%). The work that most attracts attention among the selected ones had completely different results since the object of study was the identification of stress through the classification of emotions. In this work, four levels were considered (low, median, high, and average) for five different emotions (anger, fear, sad, neutral, and happy) [26].

**Figure 8:**
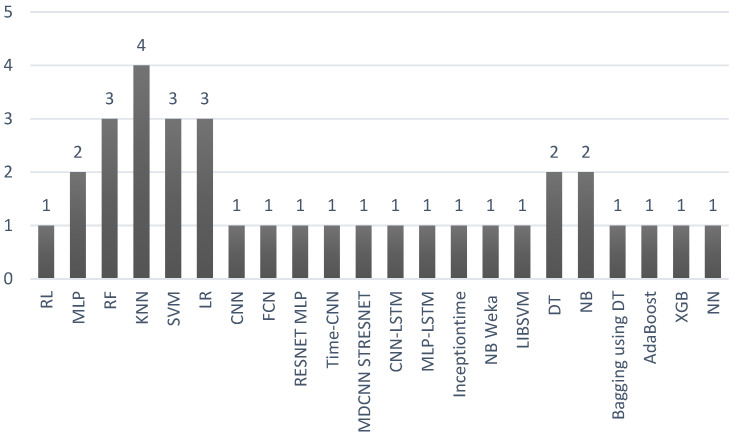
Different techniques of machine and deep learning mentioned in the reviewed literature. The X-axis concerns the machine learning and deep learning algorithms found in the investigated papers. The abbreviations are listed in the appropriate section at the end of this paper. The Y-axis is the number of times each algorithm was identified in the research works.

To answer the RQ4, we plot a graphic with all the IA techniques cited in the selected papers (Figure 7). Two papers did not employ IA in their experiments. Most investigations were based on classification problem solutions to treat the recognition of stress. As mentioned, two papers associated the emotional states and used deep learning techniques due to the multimodal data considered in their research proposals [10,26]. Figure 8 lists the acronyms of the machine and deep learning algorithms found in the research and plots the number of times each of them was mentioned in the works.

The metric used to quantify the performance of the stress-level recognition systems in most studies was accuracy. Excluding both selected papers that are not applicable to an IA metric, 70% of the results focus on accuracy comparison between the different ML or DL algorithms employed. The most complete work in terms of metrics usage was the study reported in [10], which provided the average F1-score, accuracy, and area under the receiver operating characteristic curve (ROC AUC), with the corresponding standard deviation presented in the results.

## 4. Discussion

### 4.1. Wearable Chemical Sensors

The area of wearable chemical sensors emerges as one of the main limitations of the stability of such devices, which are often exposed to uncontrolled conditions. Unlike in vitro sensing systems, which are based on controlled laboratory conditions, it is challenging to perform accurate measurements directly on the body. Wearable sensors are exposed to changing environmental and body conditions, such as variations in temperature, pH, ionic force, or humidity during prolonged indoor and outdoor activities by the device’s user [32]. Choosing the body fluid, such as sweat or interstitial fluid, to be monitored by the wearable sensor is crucial, as biomarker levels often need to be correlated with blood levels. Different metabolisms lead to different proportions between the biomarkers present in the body fluid and in the blood, which results in a challenge toward obtaining a universal correlation factor for the wearable chemical sensor in question.

The literature presents other stress-related biomarkers that can be monitored, such as the neurotransmitters norepinephrine and epinephrine, and alpha-amylase enzyme, present in blood and saliva, respectively [33]. However, the monitoring of neurotransmitters in the blood implies invasive analyses, which limits their continuous monitoring using wearable sensors. Therefore, the monitoring of stress levels is restricted to cortisol, which is present in large amounts in sweat and can be detected in a noninvasive way. The first wearable electrochemical sensor developed for the direct detection of cortisol in skin sweat was based on the development of a transistor made on a flexible substrate, with a selective molecular recognition system. The validation of such a device was performed by comparing the signals obtained by the device placed on the skin after physical exercise with those obtained by applying a cortisol spray to the skin [34]. The CortiWatch stands out as one of the main wearable chemical sensors for cortisol quantification [6]. This device uses immobilized antibodies on the sensor to selectively detect cortisol in sweat. This sensing system has demonstrated long-term stability and reliability for cortisol monitoring.

The integration of wearable chemical sensors with real-time and uninterrupted information transmission technologies is another critical factor for the consolidation of such platforms. The continuous demand for several functionalities in a sensing platform in combination with wireless communication services and data analysis increases the power requirements for the devices. Consequently, several strategies are being used to manage these energy management challenges, such as the implementation of energy harvesting techniques, the development of supercapacitors, and the manufacture of light and flexible batteries; however, device power consumption remains as one of the main problems faced by existing wearable sensors [35].

### 4.2. Critical Issues

From the 38 articles that were initially considered for full reading, only 10 articles were finally selected. The remaining were not included according to the exclusion criteria. Of the ten reported papers, two papers only were directed to analyze occupational stress. In fact, it is a rare quantitative result, and leads us to exploring new IoT solutions in this area. Moreover, just one study aimed at developing a noninvasive device for identifying the level of stress based on a chemical sensor. Based on study outcomes, developing a wrist wearable for stress identification by collecting physiological data and a chemical parameter may be challenging but possible and applicable. The related results in their investigations were promising for the use of at least three physiological biomarkers, among those that had the highest instances, such as ST, HRV, and any of GSR, EDA, or ACC. The combination of these parameters and results from this study would increase the degree of confidence in the evolution of a noninvasive wrist device regarding the level of stress, considering the challenges of occupational stress identification.

The identification of the presence or absence of stress will not present relevant or effective results. In fact, 50% of the selected works only use two levels for stress identification. Stress can lead to physiological manifestations in stress or distress situations. Stress alters homeostasis and can therefore lead to physiological, physical, and chemical manifestations, due to the presence of stress agents [36]. In this context, having more levels of stress in the identification process, up to five levels, for example, can promote better results since it allows the association of stress mitigation activities and prevents the stress level from increasing. The devices could be calibrated with the individual’s physiological information and automatically adjusts the scale in a personalized manner. Another critical point is related to the data transmission and processing employed in the investigations. Most works used a market-wearable, easy to set up, and simple-to-use device. There was missing information about data flow and storage, how IoT resources would integrate the collected data or even the process data, and how the outcomes should be employed in an IoT architecture. 

Furthermore, 60% of selected papers used Bluetooth communication protocol. This implies downloading the information through a smartphone or other gadget. For the information to be processed and synchronized, this gadget or device would preprocess the data collected, avoiding the cloud storage overload, reducing excessive communications connection, and their implications. Nevertheless, there are disadvantages associated with using this technology concerning security and data privacy. Bluetooth low energy (BLE) is the most used communication protocol for short distances, easy to find in a variety of devices such as smartphones, tablets, and notebooks, which accelerate the integration with applications. Its topology is based on master and slave devices, which create a star topology connection through pairing phases (classical Bluetooth). Despite advances in security, versions 4.0 and 4.1 (BLE) still suffer vulnerabilities to eavesdropping and man-in-the-middle attacks during pairing phases [37]. The BLE operation and phases, along with the encryption algorithms widely used in this technology, are discussed in ref. [38]. The selected papers do not consider security and data privacy in their experiments, such as encryption, identity managers, or protocol communication vulnerabilities. No issues were observed regarding the storage and treatment of data in the cloud system.

On the other hand, the selected works have employed several algorithms using machine learning and deep learning techniques based on physiological values and showed an elevated accuracy rate for identifying stress levels, although just two works ([10,26]) have already associated emotional issues in their investigations. The process of identifying emotions is a gap to be researched, and that must improve the results of the use of noninvasive wearables for this purpose [39]. A system collecting physiological parameters, a chemical sensor, and emotional recognition would increase the reliability of the stress-scale result. Despite the outcomes of the machine and deep learning techniques, the selected papers presented their outcomes from black-box algorithms and do not provide interpretability. No explainable AI algorithm was found in the included studies. Stress-level forecasting using information from patients in decision making should not be evaluated using only accuracy performance metrics [40]. 

The findings obtained in this research address important questions for the advancement of research on the use of biomarkers in the identification of occupational stress: (a) is it feasible to develop new pulse devices with digital biomarkers; (b) the use of devices available on the market for data collecting and analyzing, as is presented by the results of the study with Empatica E4, in which promise results are provided; (c) the security mechanism for devices that use Bluetooth as a communication protocol must be implemented; and (d) the use explainable artificial intelligence techniques for IoT healthcare should be considered. This review suggests a relevant limitation since there is no clear and objective definition of stress, and its assessment considers quantitative terms. According to Ref. [36], there is a lack of data and insufficient capabilities of existing modeling techniques to evaluate stress using quantitative and universal methods. Therefore, future research needs to focus on determining ways to measure and monitor stress through computational modeling while considering the personalization of the quantitative parameters.

## 5. Conclusions

When analyzing the result of this study, it is possible to identify a real potential for wearables, especially wristband (or smartwatches), in interventions that enable solutions toward the identification and control of occupational stress levels. However, obtaining robust chemical wearable sensors is still challenging, since everyone has a unique metabolism with variations in their biomarker levels, and the performance of such devices can be affected by body and environmental conditions. A promising scenario would be to develop a wearable device that allows the simultaneous monitoring of physical and chemical biomarkers and therefore provides a more accurate physiological profile. 

Chronic stress is not an isolated occurrence. It is composed of daily overloads of biochemical, physical, and emotional factors. For people who work under highly challenging conditions that generate high levels of stress, the stress will not simply disappear when the person arrives home at the end of a workday. When the stress levels persist, it can greatly affect the person’s general health and well-being. 

The workplace should be a source of health, pride, and happiness, in the sense of increasing motivation and enforcing personal development [41]. Healthy, motivated employees perform better and remain loyal to their companies for longer periods of time. When a person is constantly under a heavy workload for a long period of time and is unable to recover, their work can lead to lingering negative effects and can cause serious illnesses such as chronic stress. At the present time, combining wearable devices and parameters to identify the stress-level condition of their users become critical. Effective action can be taken early and avoid the evolution of chronic stress to burnout. Therefore, it is essential to develop more personalized recommendations, integrating them into the regular lives of people whose work brings constant emotional overload, or of people who are experiencing challenging times in their lives. The success of health managers’ efforts in the development of such technologies prioritizes a few key elements, such as identifying the population with the highest risk of developing chronic stress, accessing the correct data on this population, and creating actionable insights to guide them toward a healthier lifestyle.

## Figures and Tables

**Table 1:** Inclusion and exclusion criteria.

Inclusion Criteria	Exclusion Criteria
Articles published between 1 September 2016 and 30 September 2021	Duplicate articles
Articles with implementation results	Articles that present systematic reviews or systematic mappings
Articles published in journals and peer reviewed	Articles that do not allow open access
Articles available in their full version	Not qualifying as an article, although being classified as such in a journal (editorials, book reviews, etc.)
Articles that describe results related to stress management via smartwatches	Articles outside the scope of the search

**Table 2:** Summary of the studies selected in the systematic review.

Ref.	Type of Stress	Analyzed Information	Data Collection Time
[6]	Stress based on hormone identification	Cortisol	4 days
[10]	Stress based on the identification of emotions	Physiological signs	Data from 4 datasets
[20]	Occupational stress	Physiological signs	Data from a dataset
[21]	Stress in academic and cognitive activities	Physiological signs	50–70 min
[22]	Stress in random activities	Physiological signs	120–164 min
[23]	Occupational stress	Physiological signs	29 days
[25]	Stress in random activities	Physiological signs	7 days
[26]	Stress in academic and cognitive activities	Physiological signs	Not identified
[27]	Stress in academic and cognitive activities	Physiological signs	50 min
[28]	Stress in academic and cognitive activities	Physiological signs	Data from two datasets

**Table 3:** Characteristics of studies included in the systematic review.

Ref.	Communication Protocols	Type of Sensor	Biomarkers	ML or DL Techniques	Metrics Employed	Stress Level
[6]	Not informed	CortiWatch	Cortisol	No	Not applicable	2 levels
[10]	Not informed	Chronoamperometry	ECG, BVP, EDA, EMG, ST, Oxy, triaxial acceleration	FCN; RESNET MLP; Time-CNN; MDCNN STRESNET; CNN-LSTM; MLP-LSTM; Inceptiontime	Accuracy and other metrics WESAD: Fully Convolutional Network: 79%	2 levels
[20]	Not informed	Empatica E4	ACC, ST, BVP, EDA	NB; SVM; NN; KNN; LR; RF; DT	Accuracy	2 levels
[21]	Bluetooth	Device and chest-worn RespiBAN	HRV, ACC, ST, GSR	SVM, DT, KNN, RF, NB, ZeroR	Accuracy	3 levels
[22]	Bluetooth	Empatica E4	HRV, ST	RL	Accuracy	2 levels
[23]	Bluetooth	Wrist-worn	HRV, PPG, EDA	No	Not applicable	4 levels
[25]	Bluetooth	Device and chest-worn RespiBAN	HRV, EDA	MLP, RF, KNN, SVM, LR	Accuracy	3 levels
[26]	Not informed	Wearable wristbands with accelerometer and unspecified brand	GSR, ACC, ST	CNN	Accuracy	5 emotions levels
[27]	Bluetooth	Empatica E4	ECG, PPG, GSR	KNN, SVM, NB Weka LIBSVM	Accuracy	2 levels
[28]	Bluetooth	Empatica E4	HRV, GSR, ACC, ST	DT, RF, NB, KNN, LR, Bagging using DT, AdaBoost, XGB, MLP	Accuracy	Not mentioned

## Data Availability

Not applicable.

## Abbreviations

The following abbreviations are used in this manuscript, mentioned in the Table 3:

ACC	Accelerometers
BVP	Blood Volume Pulse
CNN	Convolutional Neural Network
CNN-LSTM	Convolutional Neural Network with Long–Short Term Memory
DA	Discriminant Analysis
DT	Decision Trees
EC	Ensemble Classifiers
EDA	Electrodermal Activity
FCN	Fully Convolutional Network
GB	Gradient Boosting
GBA	Gradient Boosting AdaBoost
GSR	Galvanic Skin Rate
HRV	Heart Rate Variability
KNN	K-Nearest Neighbor
LR	Logistic Regression
MDCNN	Multichannel Deep Convolutional Neural Network
MLP	Multilayer Perceptron
MLP-LSTM	Multilayer Perceptron with Long–Short Term Memory
NB	Naïve Bayes
Oxi	Oximetry
PPG	Photoplethysmogram
RESNET	Residual Network
RF	Random Forest
RL	Linear Regression
ST	Skin Temperature
STRESNET	Spectrotemporal Residual Network
SVM	Support Vector Machine
Time-CNN	Time Convolutional Neural Network
XGB	Extreme Gradient Boosting
